# Editorial Comment: Comparison of pain levels in fusion prostate biopsy and standard TRUS-Guided biopsy

**DOI:** 10.1590/S1677-5538.IBJU.2019.0154.1

**Published:** 2020-05-20

**Authors:** Andre Luiz Lima Diniz

**Affiliations:** 1 Instituto Nacional do Câncer Rio de JaneiroRJ Brasil Instituto Nacional do Câncer - INCA, Rio de Janeiro, RJ, Brasil;; 2 Hospital Federal da Lagoa Rio de JaneiroRJ Brasil Hospital Federal da Lagoa, Rio de Janeiro, RJ, Brasil

## COMMENT

It is overwhelming that the concern to diagnose better is being accompanied by diagnosing carefully. We see in this article the ultimate medical art that combines technological excellence and zeal with the well-being of our patients.

Following the tendency proposed by the English study PROMISS ( 1 ) authors applied the Multiparametric magnetic resonance imaging (MpMRI) in the screening scenario, using this tool for biopsy-naïve patients. Although they recognized a bias in the allocation of individuals, the evidence of a higher detection rate in the group with PIRADS ≥ 3 adds data to the literature and supports the indication of that refined imaging tool ( 2 ).

When fulfilling the objective of their study, the authors inform us about the similar pain potential of the technique under fusion of images in relation to the standard biopsy; demystifying one of the many questions about its use.

Despite all care taken by the team, their results reveal a major problem about the invasiveness of our procedures.

Let us remember the beginnings of the technique of image acquisition for MpMRI that included, pretty far behind, the use of endo-rectal coil ( 3 ). Certainly, in addition to being costly, impale (not to use more coarse terms) caused great discomfort to those who underwent that exam and diagnostic performance of MpMRI is not significantly different if endorectal coil is used or not ( 4 ). It didn’t take long, the device is no longer part of the routine of radiology clinics, making it more acceptable to patients and recommended by urologists ( 5 ).

Prostate biopsy has long been stressful for all concerned.

Hematuria and hematochezia may cause fright and fear for the patient; but for his urologist, sepsis, prostatitis and acute urinary retention are source of great unease. Several studies have researched ways to reduce infectious complications ( 6 ) and literature proposes to understand how to turn the procedure safe and comfortable for the patient ( 7 ); and for the practitioner, faster and assertive, by improving skill acquisition techniques ( 8 - 11 ).

Various anesthetic approaches have been proposed and compared ( 12 ). Although the local anesthesia routes such as intrarectal local anesthesia (IRLA) and periprostatic nerve blockade (PNB) are the most common for the urologist ( 13 ), studies have assessed the suitability of total intra venous sedation (TIVS) ( 14 ). In a prospective randomized-controlled trial (RCT), Tobias-Machado et al. ( 15 ) demonstrated that application of PNB and TIVS together were associated to higher tolerance of the exam and patient comfort. In other study, authors provided TIVS alone and demonstrated a short procedure time with sufficient analgesia, allowing patients to be discharged less than 2 hours after biopsy ( 16 ).

In a recent meta-analysis study, one of its arms evaluated the employing of sedation for transrectal prostate biopsy; evidence suggests that TIVS and PNB allows a better approach ( 13 ). In Rio de Janeiro, Brazil, at the National Cancer Institute - INCa - a branch of the Department of Urology the Prostate Cancer Diagnosis Center - CDCP - routinely performs transrectal ultrasound guided biopsies of the prostate with the support of anesthetists who promote total intravenous sedation of properly monitored patients. Despite increasing the operational cost, the implementation of advanced anesthetic management makes the procedure safe and agile; by adopting the outpatient model, CDCP increased the availability of spaces for biopsies in state’s public health system, with an installed capacity to perform 3600 procedures per year.

For most of the patients, several psychological factors, such as anxiety, make the procedure even more difficult ( 17 , 18 ). Fear and embarrassment has been described as reasons for prostate biopsy refusal ( 19 ). Although the fear of pain seems obvious, it is necessary to discuss which pain the patient has the greatest aversion to. This study sheds light on this question and leads us to believe that not the needle, but the rectal introduction of a phallic object would be the main hassle factor to that individual already weakened by their cancer suspicion.

In daily urological practice, cultural aversion to digital rectal examination (DRE) is a precursor to a number of problems for the diagnosis of prostate cancer ( 20 ). We often hear from some patients the refusal to do the DRE and the exclusive acceptance of the PSA for their screening.

Paralleling the DRE, would our patients be more resentful of the offense to their masculinity caused by the ultrasound probe or does that pain really surpass that of the needle bites suffered? Qualitative studies, with adequate discourse analysis, could help us to understand this psycho-social aspect of our role ( 21 ).

In the present paper, our authors applied the most used anesthetic approach, IRLA and PNB, and highlights the absence of difference in pain pattern of both sample harvesting ways. In a study of similar comparison, but with quite different methods, another group of investigators have shown that men undergoing targeted and systematic prostate biopsies experience more discomfort and anxiety during the procedure than those undergoing systematic biopsy alone ( 17 ). The psychological factor was evaluated in both studies, suggesting the importance of this issue; in this sense, sedo-analgesia plays an important role when used together with local anesthesia ( 22 , 23 ).

Regardless of the technique used, it is important to reduce the negative impacts that our invasive methods may cause on patients. After all, the waiting line is full and it is better that they say, “with this doctor, it didn’t hurt at all”.
